# A177 RESILIENCE AND EMPOWERMENT OF INDIGENOUS PERSONS LIVING WITH INFLAMMATORY BOWEL DISEASE: A PHOTOVOICE STUDY FROM CANADA

**DOI:** 10.1093/jcag/gwae059.177

**Published:** 2025-02-10

**Authors:** U Teucher, D Jennings, A Gannon, M Andkhoie, C Brass, S Fowler, M Johnson-Jennings, J Osei, L Porter, R Porter, R Sanderson, A P Souza Lira, J Peña-Sánchez

**Affiliations:** University of Saskatchewan, Saskatoon, SK, Canada; University of Washington, Seattle, WA; University of Saskatchewan, Saskatoon, SK, Canada; University of Saskatchewan, Saskatoon, SK, Canada; Muskoday First Nation, Muskoday First Nation, SK, Canada; University of Saskatchewan, Saskatoon, SK, Canada; University of Washington, Seattle, WA; University of Saskatchewan, Saskatoon, SK, Canada; One Arrow First Nation, One Arrow First Nation, SK, Canada; York Factory First Nation, York Factory First Nation, MB, Canada; James Smith Cree Nation, James Smith Cree Nation, SK, Canada; University of Saskatchewan, Saskatoon, SK, Canada; University of Saskatchewan, Saskatoon, SK, Canada

## Abstract

**Background:**

There is limited evidence about inflammatory bowel disease (IBD) among Indigenous peoples (Inuit, Métis, and First Nations [FN]). Healthcare utilization inequities between FN and non-FN individuals with IBD have been documented.

**Aims:**

We aimed to explore the perceptions of FN persons living with IBD, including identifying barriers and facilitators to access healthcare and understanding their expectations of healthcare for IBD.

**Methods:**

Six FN community members shared their experiences and perspectives about IBD. Four were individuals living with IBD and two were parents of FN persons living with IBD. Participants were asked to make photos in response to: “What is IBD for you?”, “What does it mean to be healthy and unhealthy when living with the diagnosis of IBD?”, “What do you expect when looking for healthcare at a clinic or a hospital?”, and “What are your challenges when accessing care?”. Then, they participated in semi-structured group reflections about their photo selections. Reflexive thematic analysis was utilized to analyze photographs, questionnaires, and reflections.

**Results:**

The analysis revealed five themes: 1. Diagnosing IBD; 2. Living with IBD; 3. The Health-Sickness Dichotomy; 4. Healthcare Enablers and Barriers; and 5. Advocacy. The 1^st^ theme encapsulates participants’ experiences regarding delays in diagnosis, the tendency of healthcare practitioners to focus on symptoms rather than the underlying disease, and the emotional responses to receiving an IBD diagnosis. The 2^nd^ theme traces participants’ experiences as they navigate a changed reality, confront issues of bodily betrayal, dignity, and resilience. The 3^rd^ theme highlights the contrasts between health and illness as experienced through physical symptoms, dietary choices, and social interactions. The 4^th^ theme captures participants’ experiences within the healthcare system, including their expectations, positive encounters, and challenges such as inadequate knowledge among some healthcare providers, negative interactions, finding adequate language and use of metaphors to speak about a stigmatizing illness, and the use of humor as a coping mechanism and a barrier. The 5^th^ theme reflects participants’ experiences advocating for themselves or relying on supportive networks to advocate on their behalf. Participants recognized the value of patients and advocates as experts in navigating their health journeys.

**Conclusions:**

Drawing on participants’ lived experiences, this study highlights the gaps in the healthcare system faced by Indigenous patients living with IBD, while revealing potential solutions to enhance their healthcare experiences and outcomes.

**Funding Agencies:**

SHRF-SCPOR

